# Fronto-limbic novelty processing in acute psychosis: disrupted relationship with memory performance and potential implications for delusions

**DOI:** 10.3389/fnbeh.2015.00144

**Published:** 2015-06-01

**Authors:** Björn H. Schott, Martin Voss, Benjamin Wagner, Torsten Wüstenberg, Emrah Düzel, Joachim Behr

**Affiliations:** ^1^Department of Psychiatry and Psychotherapy, Campus Mitte, Charité Universitätsmedizin BerlinBerlin, Germany; ^2^Leibniz Institute for NeurobiologyMagdeburg, Germany; ^3^Department of Neurology and Institute for Cognitive Neurology and Dementia Research, Otto von Guericke UniversityMagdeburg, Germany; ^4^Helmholtz Center for Neurodegenerative DiseasesMagdeburg, Germany; ^5^Department of Psychiatry and Psychotherapy, Medical School BrandenburgNeuruppin, Germany

**Keywords:** novelty, hippocampus, orbitofrontal cortex, rostral anterior cingulate cortex, ventral striatum, schizophrenia, psychosis, fMRI

## Abstract

Recent concepts have highlighted the role of the hippocampus and adjacent medial temporal lobe (MTL) in positive symptoms like delusions in schizophrenia. In healthy individuals, the MTL is critically involved in the detection and encoding of novel information. Here, we aimed to investigate whether dysfunctional novelty processing by the MTL might constitute a potential neural mechanism contributing to the pathophysiology of delusions, using functional magnetic resonance imaging (fMRI) in 16 unmedicated patients with paranoid schizophrenia and 20 age-matched healthy controls. All patients experienced positive symptoms at time of participation. Participants performed a visual target detection task with complex scene stimuli in which novel and familiar rare stimuli were presented randomly intermixed with a standard and a target picture. Presentation of novel relative to familiar images was associated with hippocampal activation in both patients and healthy controls, but only healthy controls showed a positive relationship between novelty-related hippocampal activation and recognition memory performance after 24 h. Patients, but not controls, showed a robust neural response in the orbitofrontal cortex (OFC) during presentation of novel stimuli. Functional connectivity analysis in the patients further revealed a novelty-related increase of functional connectivity of both the hippocampus and the OFC with the rostral anterior cingulate cortex (rACC) and the ventral striatum (VS). Notably, delusions correlated positively with the difference of the functional connectivity of the hippocampus vs. the OFC with the rACC. Taken together, our results suggest that alterations of fronto-limbic novelty processing may contribute to the pathophysiology of delusions in patients with acute psychosis.

## Introduction

Schizophrenia is characterized by a combination of negative symptoms such as attention deficits, blunted affect, or anhedonia, and positive symptoms that include (auditory) hallucinations and bizarre delusions. While negative symptoms typically persist chronically, positive symptoms are pronounced during psychotic episodes that last for several weeks or months. Delusions, uncorrectable beliefs not shared by others, are a hallmark positive symptom of schizophrenia (Frith, 2005). A common form of delusions that has been classified as a first rank symptom of schizophrenia by Schneider are delusional perceptions, i.e., the delusional, often self-referential, interpretation of *a priori* unimportant stimuli. Patients who experience delusional perceptions typically attribute a direct personal relevance to such stimuli. More generally, altered salience attribution is such a characteristic feature of schizophrenia spectrum disorders that re-classification as a “salience syndrome” has been suggested during recent revision processes of DSM and ICD (van Os, 2009). From a cognitive neuroscience perspective, pathological salience attribution in schizophrenia has been suggested to reflect abnormal mismatches between expectancy and percept and could be considered as pathological prediction errors (Corlett et al., 2010).

An influential model of positive symptoms suggests that developmentally dysfunction of temporal lobe limbic structures leads to impaired interactions between the prefrontal cortex (PFC) and the mesolimbic dopaminergic system, resulting in a cortical dopamine deficit, but also in inadequate subcortical dopamine release. The hyperdopaminergic state in subcortical structures gives rise to a blunted signal-to-noise ratio in prediction error coding, leading to aberrant salience attribution to irrelevant events. Such aberrant salience attributions clinically present as psychotic symptoms such as hallucinations or delusions (Heinz, 2002; Kapur, 2003; Heinz and Schlagenhauf, 2010).

Multiple brain structures have been implicated in processing of salience, i.e., the propensity of a stimulus to attract attention. A “salience network” consisting primarily of the anterior insula and the dorsal anterior cingulate cortex (dACC; Seeley et al., 2007) enables switching between different large-scale neural networks involved in task-related (i.e., externally directed processes) or self-referential (i.e., internally directed) processes, respectively (Menon and Uddin, 2010; Ham et al., 2013). Dysfunction of this network in schizophrenia has been suggested to lead to aberrant salience attribution resulting in delusions and hallucinations (Kapur, 2003; White et al., 2010). Albeit not typically considered part of the salience network, a set of fronto-limbic brain structures has been shown to respond to behaviorally salient stimuli, most notably the ventral striatum (VS), the rostral anterior cingulate cortex (rACC), the adjacent medial prefrontal cortex (mPFC), and the orbitofrontal cortex (OFC). The VS has been implicated to the processing of reward by coding reward prediction and prediction errors in a dopamine-dependent manner (Knutson et al., 2001; Wittmann et al., 2005; Pessiglione et al., 2006; Schott et al., 2008). The OFC, in addition to responding to behavioral salience of reward stimuli, has been shown to code reward value (Sescousse et al., 2010), particularly in the lateral region (Rothkirch et al., 2012). In patients with schizophrenia, structural alterations of the OFC have been reported and linked to duration of the first psychotic episode (Malla et al., 2011). At a functional level, a region within the medial prefrontal cortex (mPFC) that extends into the OFC has been shown to exhibit an abnormal salience response during reward feedback processing, and the magnitude of this atypical mPFC/OFC response correlated with severity of positive symptoms (Schlagenhauf et al., 2009). It must be kept in mind that the fronto-limbic cortices (rACC, dACC, OFC, and mPFC) show considerable degree of functional specialization that is subject to ongoing investigation. While the OFC has been implicated in salience processing and representation of value (Kahnt et al., 2010; Sescousse et al., 2010; Rothkirch et al., 2012), more medial portions of the fronto-limbic complex have been specifically associated with personal preference (Ludwig et al., 2014). This may reflect the well-replicated observation that the adjacent rACC and ventral mPFC are involved in self-referential processing (Kelley et al., 2002; Qin and Northoff, 2011).

While most neurobiological models of schizophrenia have focused on dysfunctional interactions between the PFC and the mesolimbic dopaminergic system, structural anatomical investigations have repeatedly shown hippocampal alterations that are detectable already in newly diagnosed and unmedicated patients and progress with disease duration (Honea et al., 2005; Pujol et al., 2014). The hippocampus, along with adjacent medial temporal lobe (MTL) structures, plays a critical role in explicit memory and, compatibly, patients with schizophrenia commonly show memory deficits (Boyer et al., 2007; Ranganath et al., 2008). In the healthy brain, a prominent function of the hippocampus within its multifaceted contribution to explicit memory is the detection and rapid encoding of novel stimuli in their spatial and temporal context. Patients with schizophrenia have been shown to exhibit increased distractibility by novel stimuli (Cortiñas et al., 2008), and this phenomenon has been linked to increased attention shifting towards unexpected outcomes (Núñez Castellar et al., 2012). Converging evidence from human and animal studies highlights the behavioral salience of novel stimuli, and hippocampal novelty processing has been shown to trigger mesolimbic dopamine release from ventral tegmental area (VTA) neurons via a polysynaptic pathway that encompasses GABAergic neuronal populations in the VS/nucleus accumbens (NAcc) and the ventral pallidum (Lisman and Grace, 2005). Based on the well-studied dysfunction of the mesolimbic dopamine system in schizophrenia and the ability of the hippocampus to promote subcortical dopamine release, Lisman and colleagues proposed that chronic, dysfunctional hyperactivity of the hippocampus might contribute to the pathophysiology of positive symptoms in patients with schizophrenia (Lisman et al., 2008, 2010).

**Figure 1:**
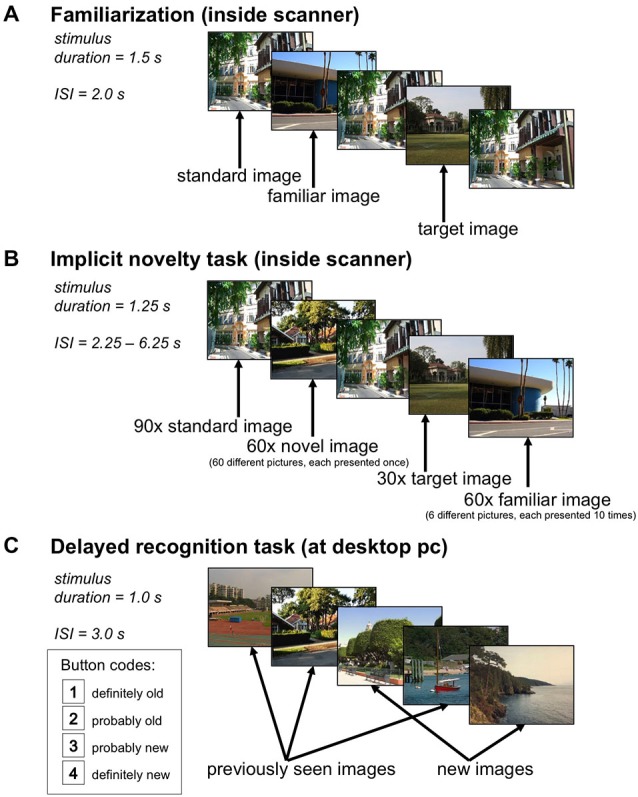
**Experimental Paradigm. (A)** In the familiarization phase, a standard image and a target image were presented 66 times each, and six additional pictures were presented ten times each, the latter ones serving as rare familiar stimuli in the main experiment. **(B)** During the implicit novelty task, 60 novel images were presented randomly intermixed with 60 familiar images (each familiar stimulus was repeated 10 times), 90 repetitions of the standard image, and 30 repetitions of the target image. Participants were instructed to respond to the target image only. **(C)** 24 h after the functional magnetic resonance imaging (fMRI) experiment, participants underwent a delayed recognition memory task in which the 60 novel images from the main experiment were presented randomly intermixed with 60 previously unseen images, and participants were instructed to respond whether or not they had seen the image on the previous day, using a four-step confidence judgment.

A fundamental limitation in the investigation of complex cognitive function in patients with schizophrenia is that most patients in clinical situations regularly take antipsychotic medication that exerts profound influence on behavioral and neural measures of cognition. On the other hand, unmedicated acutely psychotic patients often have difficulties performing more complex experimental tasks. In the present functional magnetic resonance imaging (fMRI) study, we sought to circumvent this problem by using a simple target detection task (Bunzeck and Düzel, 2006) in which participants have to respond to a previously specified target image and view all other stimuli passively (Figure 1). We hypothesized that in patients the positive relationship between the hippocampal novelty response and successful memory encoding would be attenuated (Zierhut et al., 2010). Because all patients had positive symptoms at the time of participation, we further hypothesized that they would show increased novelty-related activation of brain regions involved in salience processing and motivation, namely the striatum and fronto-limbic structures like the rACC/mPFC or the OFC.

## Methods

### Participants

Seventeen patients (13 male, 4 female) with acute psychosis were recruited from the Department of Psychiatry and Psychotherapy (Charité Campus Mitte and St. Hedwig Hospital), Charité Universitätsmedizin Berlin. All patients met ICD-10 and DSM IV diagnostic criteria of paranoid schizophrenia (ICD-10: F20.0) as assessed by at least one consultant psychiatrist. Exclusion criteria were history of neurological disorders, brain abnormalities in T1-weighted MRIs and co-morbid Axis I psychiatric disorders [Note: past depressive episodes were no exclusion criterion]. All patients were free of antipsychotic medication at the time of participation for at least four half-lives of their most recently used antipsychotic and had not used any centrally acting drugs (including benzodiazepines) for at least 12 h before participation. Psychopathological symptoms were assessed using the Scale for the Assessment of Positive Symptoms (SAPS; Andreasen, 1984a) and Scale for the Assessment of Negative Symptoms (SANS; Andreasen, 1984b). All patients exhibited positive symptoms such as delusions (mean score for “global delusions” 3.1 ± 1.0, see Table 1).

**Table 1:** **Demographic information**.

	Patients	Controls	*p*
N	16	20	
Gender (m/f)	13/3	16/4	0.740 (*X*^2^ test)
Age (± SD)	30.4 ± 6.9	31.5 ± 8.8	0.711 (*t*-test)
Educational level
Below middle school	1	0	
Middle school	5	7	
High school (*Abitur*)	10	13	0.522 (*X*^2^ test)
First language (German/other)	11/5	14/6	0.777 (*X*^2^ test)
Handedness (r/l)	15/1	19/1	0.571 (*X*^2^ test)
Smoking status (Fagerström scale 0–6)	1.38 ± 2.1	0.9 ± 1.9	0.204 (Mann-Whitney’s U-test)
SAPS/SANS—total score	93.5 ± 23.6	-	
SAPS—total score	48.2 ± 16.2	-	
SANS—total score	45.2 ± 15.3	-	
Delusions Subscore	18.5 ± 5.3	-	
*Global delusions* item	3.1 ± 1.0	-	

*SD, standard deviation; SAPS/SANS, Scale for Assessment of Positive/Negative Symptoms*.

Twenty-four healthy control subjects (20 male, 4 female) matched for age, gender, handedness, educational level (depicted by educational years) and smoking habits were recruited by public postings at the university and via Internet advertisements. Exclusion criteria in healthy controls were lifetime history of any psychiatric or neurological disorder, systemic medical illness, use of any centrally acting or illicit drugs at the time of participation, or a family history of psychosis or bipolar disorder in first-degree relatives. Patients and healthy controls performed the Brief Assessment of Cognition in Schizophrenia (BACS; Keefe et al., 2004) to evaluate their cognitive performance in different domains like verbal memory, working memory, or processing speed.

One female patient and four male healthy controls were excluded from data analysis due to excessive head movement or technical problems during data acquisition, resulting in a final study cohort of 16 patients and 20 matched healthy controls.

### Paradigm

We employed a modified version of a previously reported visual novelty paradigm (Schott et al., 2011), using the same stimulus material. Figure 1 displays the experimental setup of the task. During acquisition of the anatomical MR image used for normalization (see below), participants underwent a familiarization phase in which they viewed a total of eight photographs of outdoor scenes on a back projection screen. A standard picture and a target picture were repeated 66 times. Six additional images were repeated 10 times in a pseudo-random Latin square order (Figure 1A) and served as familiar rare items in the main task. After the familiarization phase, participants were explicitly reminded which picture was the target.

The fMRI experiment consisted of a single scanning session. Novel and familiar stimuli (photographs of outdoor scenes) were presented, randomly intermixed with a standard image and a target image (stimulus duration = 1.25 s), with an interstimulus interval (ISI) jittered between 2.25 s and 6.25 s using a near-exponential distribution, to optimize estimation of the blood oxygen level-dependent (BOLD) response (Hinrichs et al., 2000). A total of 240 picture stimuli were presented, including 90 repetitions of the standard image, 30 repetitions of the target image, 60 rare familiar scenes (the six additional pictures from the familiarization phase, each repeated 10 times), and 60 rare novel scenes (see Figure 1B). Participants were instructed to respond via button press whenever the target picture was presented, but just passively viewed all other images. The order of images was newly randomized across participants, as was the subset of novel targets, which consisted of 120 images, with the other half being used as distracters in the delayed recognition phase (see below).

Twenty-four hours after the fMRI experiment, participants performed a delayed recognition test (Figure 1C). The 60 novel targets from the fMRI experiment were presented again, randomly intermixed with 60 previously unseen photographs. Participants responded via mouse button whether or not they recognized the pictures from the previous day. False positive responses were explicitly discouraged.

### Behavioral Data Analysis

The primary behavioral variable of interest was performance in the delayed recognition memory test. To obtain measures of memory performance that account for both hits and false alarms, we computed d’ values for each subject (Stanislaw and Todorov, 1999). In three subjects (two controls, one patient), false alarm rates were 0 and were therefore adjusted by adding an error of 0.5/N (MacMillan and Kaplan, 1985). The resulting adjusted d’ values were used for brain-behavior correlations (see below).

### MRI Acquisition

MR images were acquired on a Siemens Sonata 1.5T MRI system (Siemens, Munich, Germany) using a standard head coil. 450 T2*-weighted echo-planar images [EPIs; TR = 2.0 s; TE = 35 ms; 35 axial slices (64 × 64 in-plane resolution); voxel size = 3.5 × 3.5 × 3.5 mm] were acquired in an ascending order (from bottom to top). Six volumes were acquired at the beginning of each run to allow for magnetic field stabilization and discarded from analysis. A co-planar T1-weighted MPRAGE image was acquired before the functional session and used for optimized normalization (see below).

### Functional MRI Data Processing and Analysis

Data were analyzed using Statistical Parametric Mapping (SPM8, Wellcome Trust Center for Neuroimaging, London, UK). EPIs were corrected for acquisition delay and head motion. The subjects’ individual T1-weighted MPRAGE image was then coregistered to the mean EPI and segmented using the segmentation algorithm implemented in SPM8. EPIs were then normalized into a common stereotactic reference frame (ICBM)^1^ using the normalization parameters obtained from segmentation of the MPRAGE image [voxel size: 3 × 3 × 3 mm]. Normalized EPIs were smoothed using a Gaussian kernel [FWHM = 8 × 8 × 8 mm]. A high pass filter with a cut off frequency of 128 s was applied to the data.

Statistical analysis was performed in a two-stage Mixed Effects model. In the first stage, neural activity was modeled by a delta function at stimulus onset. Event-related BOLD responses were modeled by convolving these delta functions with a canonical hemodynamic response function (HRF). The resulting time courses formed the regressors of interest (novel and familiar target stimuli, standard picture) in a General Linear Model (GLM). The six rigid-body movement parameters determined from motion correction were included as covariates of no interest, plus a single constant representing the mean over scans. GLM parameters were estimated using a restricted maximum likelihood (ReML) fit. To assess the interaction between novelty and diagnosis, single subjects’ contrast images (novel vs. familiar) were submitted to a second level random effects analysis with diagnosis as fixed factor and age as covariate. Regions of interest (ROIs) were defined anatomically for the hippocampus (based on a probabilistic localization of the CA regions and the subiculum; SPM Anatomy Toolbox; Eickhoff et al., 2005) and the striatum (anatomical automated labeling, AAL; WFU Pickatlas, Wake Forest University) and by a combined anatomical and literature-based probabilistic approach (Schubert et al., 2008; Zweynert et al., 2011) for the OFC, the rACC and the VS (the Matlab script for ROI generation and the full coordinate lists are available from the authors upon request). The significance threshold was set to *p* < 0.05, small-volume-corrected for family-wise error (FWE) within the respective ROIs. For illustrative purposes only, figures display activations at *p* < 0.005, uncorrected. Peak activations (contrasts of parameter estimates) of significant between-group differences in the hippocampus were extracted and submitted to *post hoc* correlation analyses with memory performance (adjusted d’ values) using robust Shepherd’s pi correlations (Schwarzkopf et al., 2012; see Section Results).

### Functional Connectivity Analysis

In order to assess alterations in functional connectivity of the hippocampus and OFC during novelty processing in psychotic patients relative to healthy controls, we employed the psycho-physiological interaction approach (PPI; Friston et al., 1997). PPI is defined as the change in contribution of one brain area to another with experimental or psychological context (Friston et al., 1997; Gitelman et al., 2003). Based on the critical role of the hippocampus in novelty processing and on the pronounced novelty response of the OFC in the patient group (see Section Results) we used the hippocampus and OFC as seed regions. At the single subject level, separate PPI models were computed. For each participant, the first eigenvariate time series from a sphere seeded around the voxel with the highest variance explanation within the hippocampus and OFC ROIs, respectively, were extracted and deconvolved with the canonical HRF. This combined anatomical and functional definition of the seed regions was chosen to achieve a reasonable tradeoff between anatomical specificity and signal-to-noise ratio. The resulting time series were convolved with the psychological function of novelty (novel vs. familiar rare images) and subsequently reconvolved with the HRF, yielding the new variables *X*, which were entered as primary covariates of interest into new GLMs. The original BOLD eigenvariates and the psychological variable P (novel vs. familiar) convolved with the HRF formed further covariates in the GLM design matrices. We also included the regressors of the standard and target pictures and the six movement parameters determined as covariates of no interest, plus a constant representing the mean over scans. At second level, a two-way between-subjects ANOVA (patients vs. controls × hippocampal vs. orbitofrontal seed region) with age and smoking status (Fagerström score) as covariates was computed. A small-volume FWE-corrected significance level of 0.05 was applied, correcting for combined anatomical and probabilistic ROIs of the rACC and of the VS/NAcc. In the patients, peak contrasts of parameter estimates of significant between-group differences in the rACC/mPFC were extracted and submitted to *post hoc* correlation analyses with SAPS global delusion scores using robust Shepherd’s pi correlations (Schwarzkopf et al., 2012; see Section Results).

## Results

### Behavioral Results

Table 2 displays the descriptive statistics of the target detection task and of the delayed memory task in patients and controls. During the fMRI experiment, all participants performed the target detection task with high accuracy, with no significant difference in hit rates between patients and controls (*Z* = 0.36, *p* = 0.359; two-sample Mann-Whitney U-test). Patients, however, showed a slightly higher false alarm rate (*Z* = −1.77, *p* = 0.038; two-sample Mann-Whitney U-test) and a trend for longer reaction times (*T*_30_ = −1.74, *p* = 0.046, one-tailed) [*Note*: Due to technical difficulties, behavioral data from the fMRI sessions were not available in two controls and two patients, and those subjects were excluded from these behavioral data analysis].

**Table 2:** **Descriptive statistics of the behavioral results**.

	Patients	Controls
Target detection task (fMRI)
Hits	0.983 ± 0.031	0.976 ± 0.061
RT hits	607 ± 92	565 ± 43
False alarms	0.008 ± 0.011	0.006 ± 0.021
Delayed memory task
Hits	0.23 ± 0.19	0.38 ± 0.19
False alarms	0.17 ± 0.19	0.24 ± 0.14
Adjusted d’	0.407 ± 0.423	0.438 ± 0.512

*RT, reaction time. RTs for false alarms are not reported due to low numbers. The d’ values were adjusted for false alarm rates of 0 in two controls and one patient*.

In the delayed memory task, both groups exhibited above-chance recognition performance, with hit rates being significantly higher than false alarm rates in both groups (controls: *T*_18_ = 4.91, *p* < 0.001; patients: *T*_15_ = 3.74, *p* = 0.001). There were no significant between-group differences in adjusted d’ values (*T*_34_ = 0.19, *p* = 0.850) [*Note*: Corrected hit rates (hits—false alarms) were significantly higher in the control group; *T*_34_ = 2.12, *p* = 0.041, two-tailed].

### Hippocampal Novelty Processing in Patients and Controls

**Figure 2:**
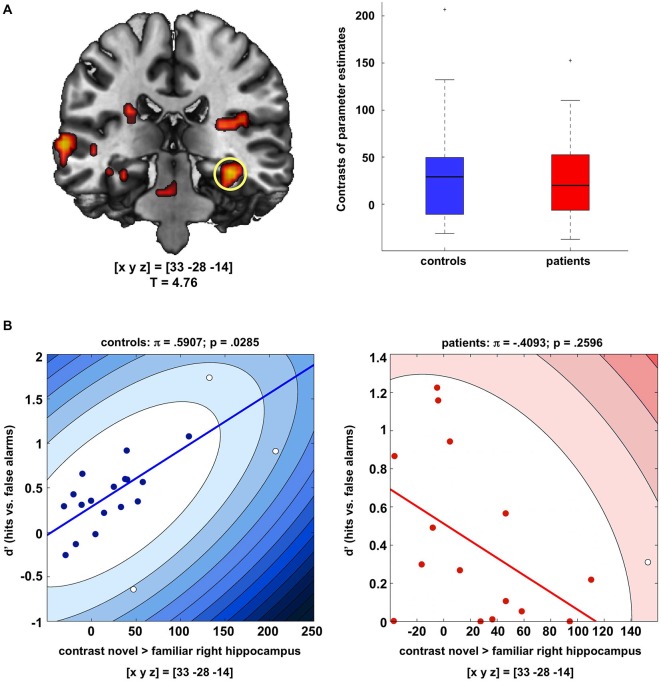
**Hippocampal novelty processing in patients and controls. (A)** Both patients and healthy controls exhibited reliable activation of the right and, to a lesser extent, also of the left hippocampus during presentation of novel as compared to familiar rare images (*p* < 0.05; FWE-corrected for anatomical hippocampus ROI). Bar plots depict contrasts of parameter estimates at peak voxels +/− standard errors. Activations are shown at *p* < 0.005, uncorrected, for illustrative purpose only. **(B)** In the healthy controls, hippocampal activation to novel images was positively correlated with successful recognition of the images after 24 h (d’ values, adjusted in participants with false alarm rates of 0), whereas no significant correlation was observed in the patients. Scatter plots depict Shepherd’s *pi* correlations, separately for controls (left) and patients (right).

Both, healthy controls and patients exhibited a robust response of the right hippocampus to novel as compared to familiar rare pictures (*p_FWE_* = 0.006), and there was no significant difference in novelty-related hippocampal activation between patients and controls (Figure 2A). We also observed a trendwise activation of the left hippocampus to novel vs. familiar rare pictures in both patients and controls ([*xyz*] = [−30 −28 −17]; *p_FWE_* = 0.051).

To assess the relationship between hippocampal activity during novelty detection and delayed memory performance, correlations were computed between the peak activation in the right hippocampus to novel vs. familiar pictures and recognition performance in the delayed memory test (d’ values, adjusted for false alarm rates of 0 in two controls and one patient), separately for patients and controls. Because brain-behavior correlations have been criticized for their sensitivity to outliers (Rousselet and Pernet, 2012; Schwarzkopf et al., 2012), we employed robust Shepherd’s *pi* correlations, in which outliers are first excluded based on the bootstrapped Mahalnobis distance, followed by a non-parametric Spearman correlation (Schwarzkopf et al., 2012). Controls exhibited a moderately strong positive correlation between novelty-related hippocampal activation and delayed recognition performance (*π* = 0.591, *p* = 0.0285, two-tailed; Figure 2B, left panel). In the patients, the correlation between hippocampal novelty responses and d’ values was not statistically significant and nominally negative (*π* = −0.409, *p* = 0.2596, two-tailed; Figure 2B, right panel). A direct comparison of the correlation coefficients of controls vs. patients using Fisher’s *Z* test yielded a highly significant difference (*Z* = 2.83; *p* = 0.0047, two-tailed) [*Note*: When using Spearman’s correlations without outlier detection, this pattern remained qualitatively unchanged (controls: *ρ* = 0.559, *p* = 0.0103; patients: *ρ* = −0.339, *p* = 0.1990)].

### Orbitofrontal Novelty Response in Patients with Schizophrenia

**Figure 3:**
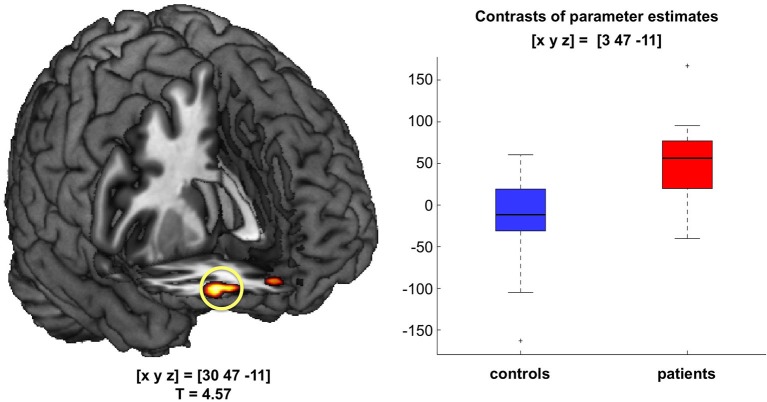
**Orbitofrontal novelty processing in patients with acute psychosis**. During presentation of novel images, patients, but not controls, exhibited robust activation of the right orbitofrontal cortex (OFC; *p* < 0.05; FWE-corrected for a combined anatomical and literature-based ROI of the OFC). Bar plots depict contrasts of parameter estimates at peak voxels +/− standard errors.

In a direct comparison of patients’ and controls’ novelty contrasts, patients showed a robust fMRI response in the right OFC to novel vs. familiar rare pictures that was absent in healthy controls (Figure 3). Similar to the novelty response in the novelty-related hippocampus, novelty-related OFC activation in patients did not significantly correlate with delayed recognition memory performance (adjusted d’ values; *π* = 0.1254, *p* = 1.0).

### Novelty-Related Hippocampal and Orbitofrontal Functional Connectivity

**Figure 4:**
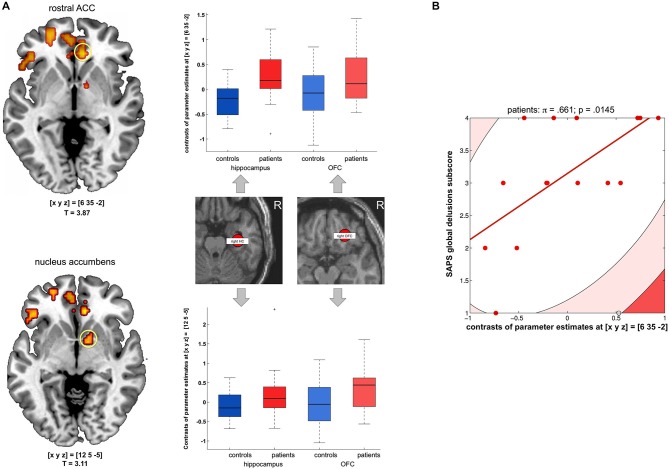
**Functional connectivity of the hippocampus and OFC to the rACC. (A)** Left panel: Patients exhibited stronger novelty-related functional connectivity from both the hippocampus and OFC to the rostral anterior cingulate cortex (rACC, top) and to the nucleus accumbens (NAcc, bottom); activations are displayed at *p* < 0.005, uncorrected for illustrative purposes only. Right panel: Box plots display contrasts of parameter estimates of novelty-related functional connectivity (median +/− 25 percent quantiles, separated by group and anatomical seed region) to the rACC (top) and NAcc (bottom). All *p* < 0.05, small-volume FWE-corrected for a combined anatomical and probabilistic rACC ROI (Holtmann et al., 2013). **(B)** Scatter plot depicting Shepherd’s *pi* correlation of the difference between hippocampal and orbitofrontal connectivity (contrasts of parameter estimates) and global delusion subscores of the scale for the assessment of positive symptoms (SAPS) (applicable to patients only). The correlation survived Bonferroni correction for the two correlations computed (sum and difference of the contrasts of parameter estimates reflecting hippocampal and orbitofrontal connectivity to the rACC).

To further investigate potential neural networks underlying the OFC novelty response in the patients, we computed a functional connectivity analysis using the PPI approach with the hippocampus and OFC as seed regions and novelty vs. familiarity as psychological variable (see Section Methods for details). While there was no direct novelty-related functional connectivity increase between the hippocampus and OFC in the patients, both seed regions exhibited increased novelty-related functional connectivity with the rACC in patients when compared to healthy controls (*p* = 0.015, FWE-corrected for ROI volume; 2 × 2 random effects ANOVA model, T-test-based comparison of hippocampal and OFC PPI contrasts; see Figure 4A). This finding suggests that the rACC might function as a hub linking hippocampal and OFC novelty responses in the patients. To test whether this increased functional connectivity of the hippocampus and OFC with the rACC might be related to delusions, we computed Shepherd’s *pi* correlations of the sum and the difference of their contrasts of parameter estimates and the global delusions subscale of the SAPS. While there was no significant correlation of the sum, the difference (hippocampal—orbitofrontal connectivity to the rACC), was positively correlated with the global delusions score in the patients (*π* = 0.661; *p* = 0.0290, two-tailed, Bonferroni-corrected; see Figure 4B).

A significant connectivity increase of both the hippocampus and the OFC in the patients was observed in the VS/nucleus accumbens (small-volume FWE-corrected *p* = 0.033; see Figure 4A, bottom). However, this functional connectivity increase did not correlate with global delusion scores (all *p* > 0.290).

## Discussion

Our results provide further evidence for hippocampal dysfunction in schizophrenia, as evident from the absent relationship between the hippocampal novelty response and long-term memory performance. Moreover, patients exhibited a novelty-related OFC activation that was absent in the controls. Functional connectivity results further suggest that acute psychotic states might be accompanied by an processing of novel stimuli in fronto-limbic structures.

### Disrupted Relationship Between Hippocampal Novelty Processing and Memory Performance in Schizophrenia

Patients, like healthy controls, exhibited a robust hippocampal response to novel vs. familiar stimuli, suggesting a preserved hippocampal novelty response in acute psychosis. The relationship between novelty-related hippocampal activation and successful memory encoding was, however, disrupted in the patients. While the hippocampal novelty response was positively correlated with later recognition memory performance in the control cohort, no such relationship was observed in patients. Several previous studies in healthy participants (Hariri et al., 2003; Bertolino et al., 2006; Schott et al., 2011, 2014; Barman et al., 2014) and neurological patient populations (Oedekoven et al., 2014; Hulst et al., 2015) have suggested a positive relationship between fMRI activation of the hippocampus during novelty processing or encoding and memory performance. In patients with schizophrenia, however, that relationship appears to be disrupted. For example, patients with schizophrenia exhibit a modulation of hippocampal activity by level of processing (LoP), but that hippocampal activation in the patients does not predict memory performance (Ragland et al., 2006a,b; Zierhut et al., 2010). Our results expand those observations by showing, similar to LoP, stimulus novelty does elicit a hippocampal neural response, but this response is not associated with successful encoding, and the present data therefore suggest a disruption of the relationship between hippocampal novelty responses and memory formation in patients with acute psychosis. While the lack of such a correlation in the patients may not be considered a deficit *per se*, it might nevertheless provide further—at least indirect—evidence for the previously suggested role of the hippocampus in the pathophysiology of schizophrenia. As discussed by Lisman et al. (2008, 2010), hippocampal pyramidal cells are most likely overactive in patients with schizophrenia. While tonically increased activity of the hippocampus might not be readily detectable by means of activation-based fMRI, it is conceivable that a decreased signal-to-noise ratio resulting from such increased tonic activity may attenuate the relationship between hippocampal BOLD signal and successful memory formation. In a recent high-field fMRI study, it could be demonstrated that, in healthy humans, hippocampal input structures (dentate gyrus, CA3, apical CA1) primarily respond to novelty while successful encoding has been associated with activation of output structures (pyramidal CA1, subiculum; Maass et al., 2014). Notably, computational models of MTL pathology suggest that deficits in recall-based and particularly in context-specific memory processes in patients with schizophrenia might result from the disruption of intra-MTL connectivity (Talamini et al., 2005; Talamini and Meeter, 2009). In the present study, a 1.5T MR tomograph was employed, and the spatial resolution of our EPIs did not allow for a differential investigation of hippocampal subfields. We can therefore only tentatively suggest that in unmedicated patients with schizophrenia there might exhibit a disruption of intra-hippocampal functional connectivity at the level of either hippocampal output regions or intra-hippocampal neural circuitry.

A recent influential model suggests that chronic disinhibition of hippocampal pyramidal cells and resulting overstimulation of mesolimbic dopaminergic nuclei by a polysynaptic pathway originating in the hippocampus might trigger positive symptoms (Lisman et al., 2008, 2010). Hippocampal novelty detection has been suggested to elicit increased stimulation of the NAcc by the hippocampus, which in turn reduces the tonic inhibition of the VTA by the ventral pallidum, ultimately promoting dopamine release in the NAcc and hippocampus (Lisman and Grace, 2005). In healthy humans, novelty detection has been linked to co-activation of the hippocampus and dopaminergic midbrain (Schott et al., 2004; Bunzeck and Düzel, 2006). In a series of studies, it could be further demonstrated that moderate enhancement by either reward-related enhancement of endogenous dopaminergic activity (Bunzeck et al., 2009) or pharmacological stimulation of dopamine release via the dopamine precursor L-dopa (Eckart and Bunzeck, 2013) elicits accelerated novelty processing in healthy humans, while the presumably further increased dopamine release by combination of reward and L-dopa has been associated with delayed novelty signals and impaired recognition (Apitz and Bunzeck, 2013). One potential explanation for this oberservation might be an inverse U-shaped function of dopaminergic action in the hippocampus, similar to the well-characterized modulation of prefrontal function by dopamine levels (Meyer-Lindenberg and Weinberger, 2006). Moreover, an imbalance of tonic vs. phasic dopaminergic activity also differentially affects memory performance with pharmacologically enhanced phasic dopaminergic activity being associated with improved performance while increased tonic dopaminergic stimulation has the opposite effect (Knecht et al., 2004; Breitenstein et al., 2006). Given the well-documented increased presynaptic dopaminergic activity (Bonoldi and Howes, 2013) and the dysregulation of tonic vs. dopamine action (Goto et al., 2007) in schizophrenia, dysfunction of the dopaminergic system might constitute an additional pathomechanism underlying the disrupted translation of hippocampal novelty signals into successfully encoded engrams.

One limitation of the present study is that, while we did observe a robust hippocampal signal to novel stimuli in both, controls and patients, no midbrain activation was found in either group. The most likely reason for this is that the study was conducted on a 1.5T MR tomograph, and most studies in which midbrain activity could be reliably detected had employed field strengths of at least 3T, providing an inherently higher signal-to-noise ratio and higher spatial resolution (Bunzeck and Düzel, 2006; D’Ardenne et al., 2008; Schott et al., 2008; Krebs et al., 2009a,b). An additional—or alternative—explanation might be that, given the absence of a direct co-activation of the hippocampus and the striatum, including the NAcc, the stimuli might not have been sufficiently salient engage the hippocampal-VTA loop, at least in the control group. In the patients, on the other hand, an indirect activation might have occurred, as indexed by the functional connectivity increase between the hippocampus and OFC on the one hand and the striatum on the other.

### Fronto-Limbic Novelty Processing and its Potential Role in Delusions

While novelty-related hippocampal activation in patients did not correlate with later memory performance, the patients showed more pronounced activation and functional connectivity increases to stimulus novelty in fronto-limbic structures, most prominently in the OFC (Figure 3). The human OFC is functionally strongly connected with the mesolimbic dopamine system (Gurevich et al., 1997; Cole et al., 2012). OFC activation in response to novelty has previously also been observed in healthy humans (Bunzeck et al., 2012), and it has been linked to explicit, voluntary encoding of information into long-term memory. In a previous study using the same stimulus material, we had also observed an orbitofrontal novelty response in healthy humans (Schott et al., 2011), but, importantly, in that study, participants had performed an explicit novelty/familiarity decision, novelty was thus a task-relevant feature. Here, on the other hand, novel stimuli were processed implicitly while participants focused on the detection of a target stimulus. The OFC has been implicated in the processing of salient information, for example by coding reward value (Kahnt et al., 2010; Rothkirch et al., 2012), although other studies have suggested that the OFC and adjacent mPFC are primarily involved in conveying a more general, probability-related salience signal, while the actual value is coded by the VS (Knutson et al., 2005).

One apparently straight-forward and plausible explanation of the novelty-related OFC activation in the patient group might therefore be that patients might attribute atypical salience to the novel, but task-irrelevant stimuli, which would be in line with the previously demonstrated attention orienting towards novel stimuli in patients with schizophrenia (Cortiñas et al., 2008; Núñez Castellar et al., 2012). In a study of feedback processing in patients with schizophrenia, abnormal activation of the ventral mPFC to negative feedback in a monetary incentive delay (MID) task, and the mPFC activation during feedback processing was correlated with severity of delusions in the patient group (Schlagenhauf et al., 2009). No such correlation, however, was found in the present study, but instead, the SAPS global delusions score correlated positively with the difference of the hippocampal vs. orbitofrontal functional connectivity to the rACC/mPFC (Figure 4B). We therefore suggest that, in our study, the OFC activation to novelty observed in patients is unlikely to directly reflect dysfunctional salience processing leading to delusions. Recent studies point to considerable functional specialization within the OFC (Kahnt et al., 2012) and suggest that OFC subregions might be differentially involved in coding implicit overall salience and value of a stimulus (Rothkirch et al., 2012). Specifically, activation of the medial OFC—close to the region where a correlation between atypical feedback responses and delusions was observed by Schlagenhauf et al. (2009)—was associated with implicit salience, irrespective of value, whereas a region in the right lateral OFC—close to the OFC region in which patients exhibited a novelty response in the present study—showed a parametric modulation of activity by stimulus value. In the present study, novel and familiar distracter stimuli did not differ in terms of motivational value or semantic content, making a higher (extrinsic) motivational value of the novel stimuli unlikely. We therefore suggest that the OFC novelty response and the novelty-related functional increased connectivity between the OFC and rACC might reflect a more intrinsic stimulus evaluation process in the patients.

When stimulus evaluation in the OFC is impaired, for example as a result of psychosis-related structural alterations (Malla et al., 2011), there might be an imbalance of hippocampal vs. orbitofrontal functional connectivity with the rACC. The observation that the relative novelty-related functional connectivity of the hippocampus vs. the OFC with the rACC correlated positive with delusions raises the possibility that hippocampal-rACC interactions during processing of novel stimuli might contribute to the pathophysiology of delusions, while novelty processing within the (lateral) OFC might actually confer a protective effect. The rACC/mPFC is part of the so-called Default Mode Network (DMN) that has been implicated in social and self-referential processing (Gusnard et al., 2001) and has been specifically been linked to self-reference (Kelley et al., 2002). Erroneous self-attribution is a hallmark feature of delusions in schizophrenia and particularly of delusional perceptions (Bovet and Parnas, 1993). Patients with schizophrenia and people at high risk for psychosis exhibit impaired deactivation of the DMN (Landin-Romero et al., 2015) and fail to suppress the rACC/mPFC during task that involve no self-reference (Pauly et al., 2014; Falkenberg et al., 2015). Moreover, MTL activation during self-referential processing in patients with schizophrenia has been shown to correlate with positive symptoms (Pauly et al., 2014). Together with our present results, these data raise the possibility that dysfunctional interactions of the MTL and rACC/mPFC might give rise to aberrant self-attribution of stimuli, which may manifest clinically as delusions.

Future studies are warranted to further characterize the functional parcellation of fronto-limbic structures within this network. With respect to the OFC region that was found activated to novel stimuli in the present study, we can thus far only speculate that it could reflect an evaluation process of the stimuli that might to some extent moderate the hippocampal-rACC interactions, possibly by computing stimulus value (Rothkirch et al., 2012).

### Functional Connectivity of the Ventral Striatum

Hippocampal novelty processing has previously been linked to stimulation of dopamine release by VTA neurons (Lisman and Grace, 2005), and a hyperactive hippocampal-VTA loop has been suggested to be involved in the generation of psychotic symptoms (Lisman et al., 2008, 2010). In addition to the rACC, the VS also exhibited increased functional connectivity with the hippocampus and OFC in the patients (Figure 4A, bottom). Ventral striatal activation during reward processing has been shown to correlate with dopamine release (Schott et al., 2008), and it appears plausible that novelty-related stimulation of dopamine release in the patients might be involved in the generation of an abnormal response salience attribution. This notion is well in line with our predictions as we had hypothesized that patients in an acute state of psychosis might attribute abnormal salience to novel, but otherwise irrelevant stimuli, which might be a putative neural basis for delusional phenomena. However, unlike MTL vs. OFC to rACC functional connectivity, the connectivity between these structures and the NAcc did not correlate with psychopathology in the present study, and the behavioral relevance of the increased ventral striatal functional connectivity in the patients cannot be conclusively resolved by the present study.

## Conclusion

The present study suggests that patients in an acute psychotic state exhibit atypical processing of novel information in two ways. First, the hippocampal novelty response is decoupled from successful hippocampus-dependent encoding of the novel information into episodic memory. Second, patients exhibited a specific novelty response in the OFC and increased novelty-related fronto-limbic functional connectivity. With the hippocampal-rACC functional connectivity showing a positive correlation with delusions, our results highlight the possibility that delusions might arise from abnormal processing of novel stimuli in fronto-limbic cortices.

## Conflict of Interest Statement

The authors declare that the research was conducted in the absence of any commercial or financial relationships that could be construed as a potential conflict of interest.

## Footnotes

^1^http://loni.usc.edu/ICBM/
